# Long-term radiological and clinical outcomes after surgical treatment of non-union: a ten-year follow-up study

**DOI:** 10.1007/s00068-026-03315-0

**Published:** 2026-08-31

**Authors:** Jessica C. Böpple, Jonas Berger, Thomas Ferbert, Tim Niklas Bewersdorf, Gerhard Schmidmaier, Sebastian Findeisen

**Affiliations:** 1 Heidelberg Trauma Research Group, Clinic for Trauma and Reconstructive Surgery, Center for Orthopedics, Trauma Surgery and Spinal Cord Injury, Schlierbacher Landstraße 200a, D-69118 Heidelberg, Germany; 2 Faculty of Medicine, Heidelberg University, Heidelberg, Germany

**Keywords:** Non-union, Long-term outcome, Bone remodelling, Lane-Sandhu-Score, SF-12

## Abstract

**Purpose:**

Non-unions are a major challenge in trauma surgery. They are known to affect patients’ lives for years after the initial trauma. Nevertheless, most studies only examine the first two years after treatment. The question what the long-term results look like ten years after surgery was posed in light of observations made in everyday clinical practice. To answer this, our study was designed to examine all patients who underwent non-union surgery in our department ten years ago.

**Methods:**

All patients who underwent surgery between July 2014 and October 2015 were invited to participate in the study. During a one-site appointment, an X-ray image was taken, which was then used to assess consolidation. The patients underwent a physical examination and answered a questionnaire, covering topics such as pain on a visual analogue scale (VAS) and overall quality of life with the short-form 12 (SF-12).

**Results:**

A total of 54 patients were included in the study. The median follow-up period was 122 months (IQR: 118-124). After 10 years 88.9% patients showed osseus consolidation with a median Lane-Sandhu-Score (LSS) of 4 (IQR: 3–4). The Wilcoxon signed-rank test revealed a significant difference compared to 24 months postoperatively (Z = 4.716, p < 0.001). The difference in the consolidation showed a marginally significant result in the McNemar test with p = 0.063. A total of 51 patients (94.4%) reported being satisfied with the overall treatment. Patients had an average SF-12 mental score of 53.14 ± 10.13 and an SF-12 physical score of 38.93 ± 12.71 after ten years. Compared to the values after 12 months postoperatively, stable values without significant changes were observed.

**Conclusion:**

The study showed that bone remodeling can still be observed many years after surgical treatment of non-union. Furthermore, it was shown that the clinical results remain stable even after ten years.

**Clinical trail number:**

DRKS00037034, registered 12.06.2025.

## Introduction

Non-unions represent a challenging complication in trauma and orthopedic surgery. In our department, we adhere to the definition of non-union established by the European Society of Tissue Regeneration in Orthopedics and Traumatology (ESTROT) [1]. According to this definition, a non-union is characterized by the absence of fracture healing after a minimum of six months, accompanied by clinical signs (pain and motion at the fracture site) as well as radiological findings (lack of bone continuity and insufficient stability) [2]. This definition serves as the basis for diagnosis and therapeutic decision-making.

Epidemiological data indicate an overall non-union rate of approximately 1.9% per fracture [3]. However, substantially higher rates have been reported for specific anatomical regions and age groups, exceeding 8% among patients aged 35- to 44-years with clavicle or scapula fractures [3]. For tibial shaft fractures, the reported non-union rate is 6.8% per fracture [4]. In Germany, 11,840 cases of non-unions were treated in 2019, corresponding to an incidence of 17.4 cases per 100,000 residents [5]. These figures highlight not only the frequency but also the clinical relevance of non-unions.

Previous studies have shown that non-unions impose a considerable burden on affected individuals and constitutes a substantial socioeconomic challenge, with median treatment costs in Germany exceeding €100,000 per patient [6].

Furthermore, there is evidence that patients without bone consolidation often suffer from long-term effects for many years after treatment. Wichlas et al. showed that even 5 years after treatment for tibial non-union, quality of life was significantly reduced compared to the general population, even in cases where treatment was successfully completed, quality of life was significantly lower than in the general population [7]. Wagner et al. also report reduced general and physical function compared to the normal population, with an average follow-up period of 8.6 years after non-union surgery [8]. 

The current literature on long term follow-up after surgical treatment of non-unions remains limited. Existing studies have primarily focused on the time to osseous consolidation or the onset of clinically relevant improvement. The aim of the present study was to provide a long-term assessment of radiological and clinical outcomes ten years after surgical treatment and to evaluate how these findings differ from those observed at earlier follow-up timepoints.

## Materials and methods

### Patients

All patients who had undergone surgical treatment for non-union at our department approximately ten years ago were identified using the hospital information system. A total of 112 patients who had received at least one surgical procedure between July 1, 2014, and October 31, 2015, were eligible for inclusion.

The surgical techniques used ranged from simple dynamic adjustment of the intramedullary nail to osteosynthesis via plate and the two-stage Masquelet procedure with the addition of Vitoss, RIA and/or BMP-2. Only patients who were of legal age at the time of surgery were considered. A radiological follow-up at 2 years was not mandatory for study inclusion. Non-unions following pathological fractures due to cancer-related bone lesions as well as patients in whom cement spacers were used as definitive bone substitution were excluded.

The study was approved by the Ethics Committee of the Medical Faculty of Heidelberg University (S-149/2025) and was conducted in accordance with principles outlined in the last version of the declaration of Helsinki. Written informed consent was obtained from all participating patients. The study was registered in the “Deutsches Register Klinischer Studien” (DRKS) with the trial number DRKS00037034.

### Postoperative assessment

Clinical as well as radiological follow up examinations accordingly to our clinical practice were performed. During these, the following variables were recorded: age, sex, body mass index (BMI), comorbidities, type of surgical procedure, anatomical location of the non-unions, and presence of infection. Patients with a Body Mass Index (BMI) of 30 or higher were considered obese. Smoking status was categorized using the threshold proposed by Kruk et al., with ≥ 6.1 pack-years defined as an increased risk for non-union [9].

Preoperative general health status was assessed using the American Society of Anesthesiologists (ASA) classification. The initial fracture was classified as open, closed or iatrogenic.

Physical examination included assessment of the range of motion (ROM) of the adjacent joints, which was compared with physiological reference values.

Pain intensity was measured using a visual analog scale (VAS) [10], and health related quality of life was assessed using the Short-Form-12 (SF-12) questionnaire [11].

Patients were additionally asked about subjective pain perception, use of analgesic medication, perceived strength deficit compared with the contralateral side, ability to achieve full weight bearing, and maximum walking distance.

### Radiological evaluation

Standardized radiographs of the affected region were obtained in two planes at follow-up.

**Fig. 1:**
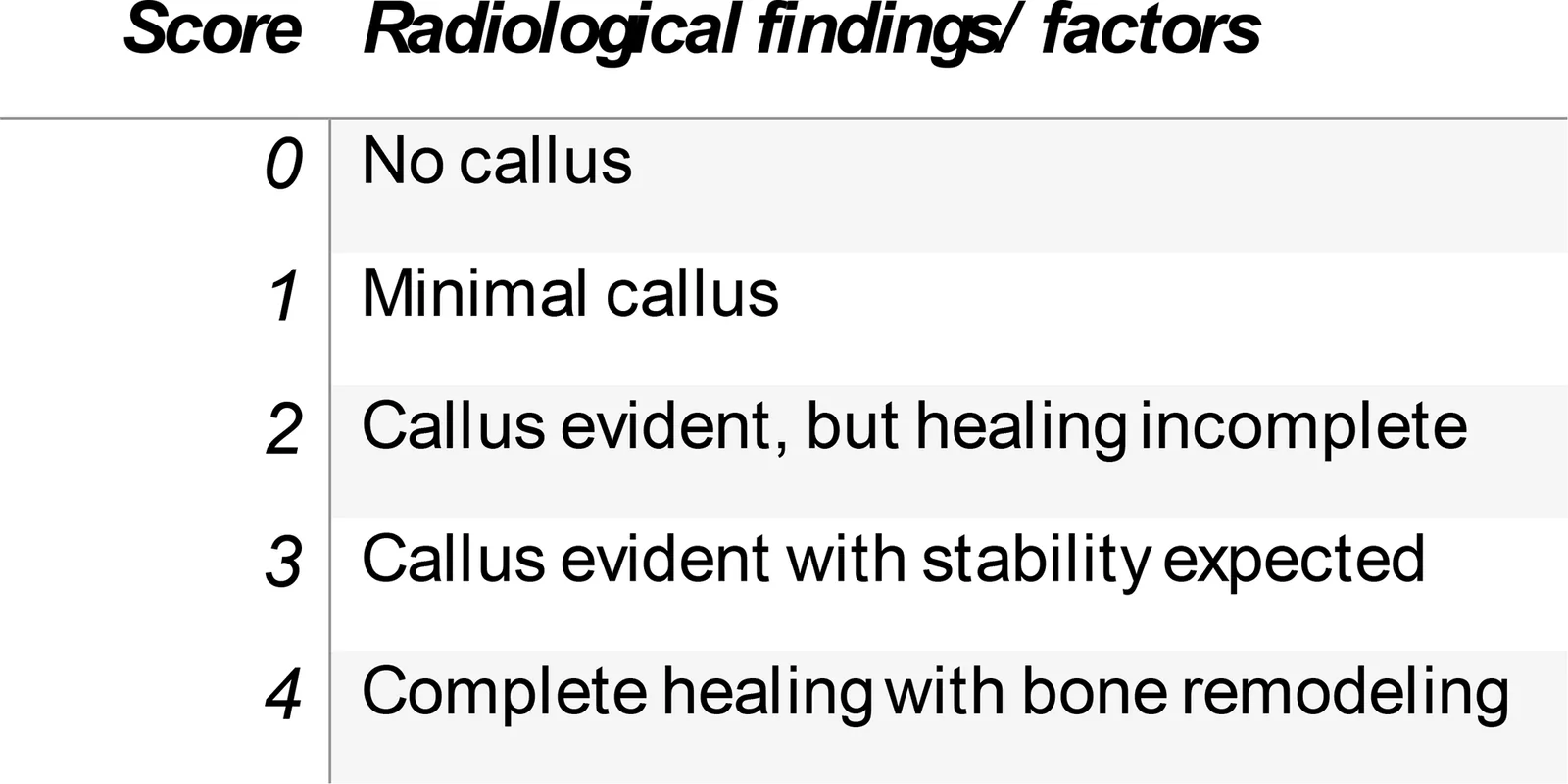
Modified Lane Sandhu Score

Bone healing was assessed using the modified Lane-Sandhu Score (LSS), which grades radiological consolidation on a scale from 0 to 4, where 4 indicates complete healing with full cortical remodeling and 0 indicates absence of healing [12]. For patients who underwent amputation, compound osteosynthesis or revision surgery and were therefore classified as treatment failures, an LSS of 0 was applied. If the 2-year radiological follow-up was unavailable, the most recent available radiological examination was used for the analysis. A detailed description of the scoring system is shown in Fig. 1. LSS values at 6 months, 12 months, 24 months, as well as 10 years postoperatively were determined independently by three consultants experienced in non-union therapy.

### Statistical analysis

The statistical analyses were performed using IBM SPSS Statistics (Version 29.0.2.0; IBM Corp., Armonk, NY, USA) as well as Stata statistical software (version 18.0, StataCorp, United States). Continuous variables were tested for normal distribution using the Shapiro-Wilk test. Descriptive data are reported as mean ± standard deviation (SD) for normally distributed data and as median with interquartile range (IQR) for non-normally distributed data. Categorical variables are presented as absolute numbers and percentages.

For comparisons between independent groups, the independent t-test was used for normally distributed continuous variables, while non-normally distributed variables were analyzed using the Mann-Whitney U test.

For comparisons of paired data within the same patient cohort at different time points, the paired t-test was used for normally distributed variables, while non-normally distributed variables were analyzed using the Wilcoxon signed-rank test.

The changes between time points were presented using cross-tabulation analysis for paired data, which allowed for the calculation of individual improvements in patients, such as from LSS 3 to 4 or from LSS < 3 to ≥ 3. The statistical significance of the changes between two and ten years was additionally tested using the Wilcoxon signed-rank test for paired ordinal-scaled data.

Categorical variables were compared using the chi-square test or Fisher’s exact test, depending on the expected cell frequency.

A multivariable logistic regression model was used to assess the association between patient-related factors and consolidation. The dependent variable was consolidation. Independent variables included BMI, diabetes, infection, and smoking (using the smoking status as defined above). For this model the BMI was categorized into two groups with one group being normally weighted with an BMI between 18.5 kg/m^2^ and 25 kg/m^2^ and the second group with every value above or under this range.

A p-value < 0.05 was defined as statistically significant.

## Results

**Fig. 2:**
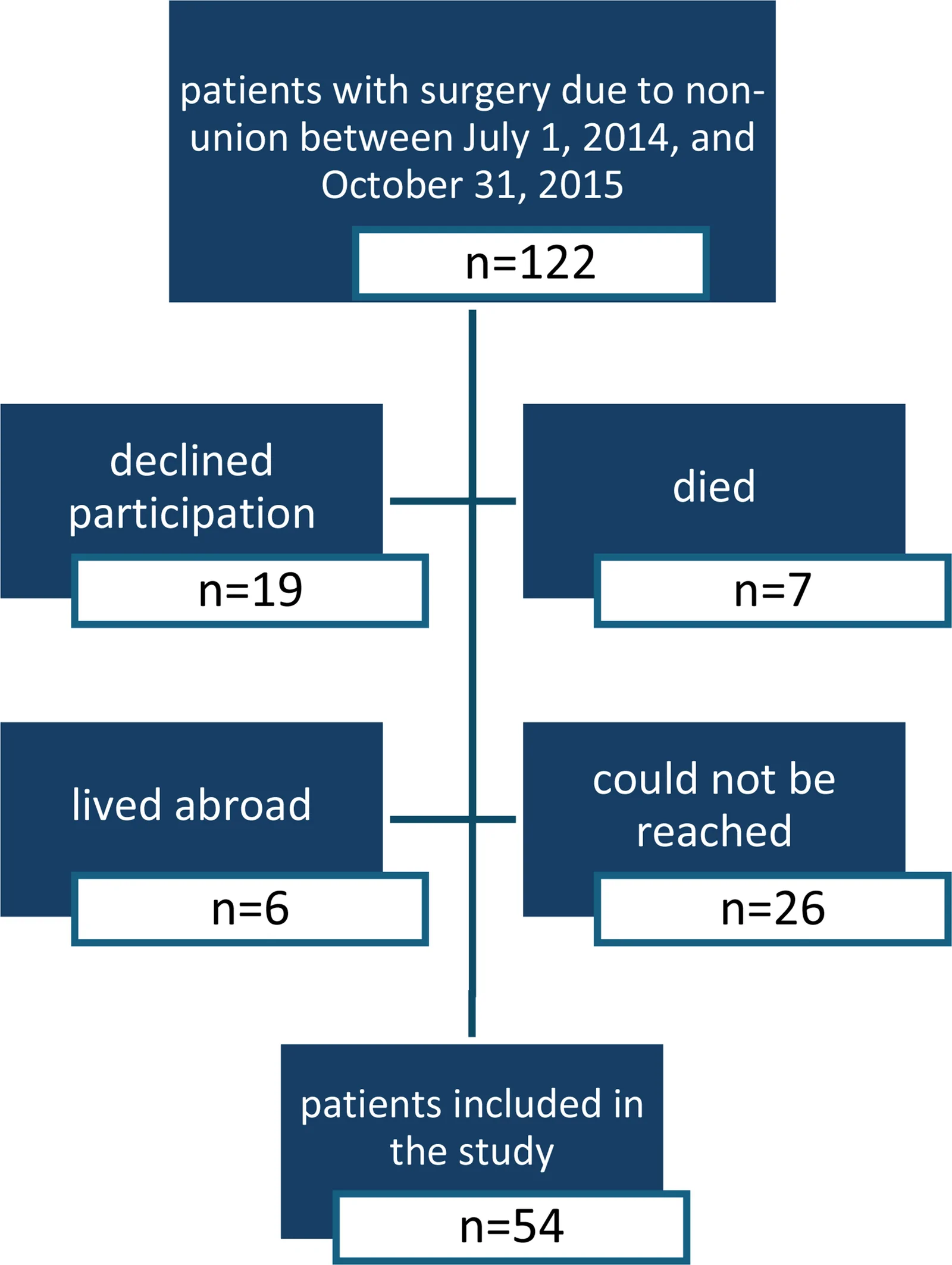
Patient recruitment and reasons for exclusion

A total of 112 patients underwent surgical treatment for non-union in our department between July 1, 2014, and October 31, 2015. Of these, 19 patients declined participation, 7 had died, 6 were living abroad, and 26 could not be reached. The remaining 54 patients were included in the study (Fig. 2). Follow-up examinations were conducted between January 1 and May 12, 2025. The median follow-up duration was 122 months (IQR: 118–124). Of the 54 patients, 34 underwent a two-stage procedure; 33 of these were treated using the Masquelet technique, while in one patient, the existing metal was removed arthroscopically during the first procedure and new osteosynthesis material was inserted during the second.

The remaining 20 patients were treated with a single operation for non-union. In 17 patients, this involved reosteosynthesis; in one patient, it involved dynamization of the embedded intramedullary nail; in one, debridement with the addition of Vitoss, RIA, and BMP-2; and in one, debridement and metal removal.

The 54 included patients had a median age of 52 years and consisted of 15 women and 39 men. The lower extremity was affected in 47 patients and the upper extremity in 7 patients. A smoking history of ≥ 6.1 pack years was reported in 26 patients.

In this cohort, the median of the preoperative LSS was 1 (IQR: 0; 1). A total of 23 patients had a value of 0, 28 had a value of 1, and 3 had a value of 2.

Additional baseline characteristics are presented in Table 1.

**Table 1:** Patient characteristics

Factor	Value
**N**	54
**Age** median in years (IQR)	52 (44–59)
**Gender** Female / Male	15 (28%) / 39 (72%)
**Follow-up** in months (IQR)	122 (118–124)
**BMI** median in [kg/m^2^] (IQR)	29 (26–33)
**Smoker** Yes / No More than 6,1 pack years - Yes / No	34 (63%) / 20 (37%) 26 (48%) / 28 (52%)
**Localization** Femur / tibia / humerus / forearm / metatarsals / clavicle	23 (43%) / 23 (43%) / 3 (6%) / 3 (6%) / 1 (2%) / 1 (2%)
**ASA-Score** I / II / III	14 (26%) / 26 (48%) / 14 (26%)
**Infection** Yes / No	18 (33%) / 36 (67%)
**Surgical-procedure** one - step / two- or multi – step	20 (37%) / 34 (63%)
**Initial fracture** Closed / Open / Iatrogenic	35 (65%) / 15 (28%) / 4 (7%)
**previous operations on the non-union** median (IQR)	3 (2–4)
**Addition of** BMP - Yes / No TCP - Yes / No Bone graft - Yes / No Spongiosa - Yes / No Bioactive glass - Yes / No	32 (59%) / 22 (41%) 25 (46%) / 29 (54%) 5 (9%) / 49 (91%) 45 (83%) / 9 (17%) 1 (2%) / 53 (98%)
**pre-existing conditions** Heart Disease - Yes / No Diabetes - Yes / No Kidney Disease - Yes / No Tumor - Yes / No Increased Cholesterol - Yes / No Thyroid Disease - Yes / No Respiratory Disease - Yes / No Osteoarthritis - Yes / No Back Pain - Yes / No Mental Illness - Yes / No	37 (69%) / 17 (31%) 12 (22%) / 42 (78%) 5 (9%) / 49 (91%) 6 (11%) / 48 (89%) 19 (35%) / 35 (65%) 10 (19%) / 44 (81%) 10 (19%) / 44 (81%) 30 (56%) / 24 (44%) 34 (63%) / 20 (37%) 13 (24%) / 41 (76%)

BMI body mass index; BMP bone morphogenetic protein; TCP tricalciumphosphate, IQR: interquartile range

### Radiological outcome

After two years, 43 out of 54 had reached osseus consolidation (LSS > 2), resulting in 79.6% and after ten years in 88.9% with 48 out of 54 patients. Over a period of 2 to 10 years, the LSS score improved from 3 to 4 in 20 of 29 patients (69.0%), indicating complete cortical remodeling. Five patients (9.3%) achieved additional consolidation over the long term, while no patient experienced a deterioration.

The difference of consolidation between 2 and 10 years showed a marginally significant result in the McNemar test with *p* = 0.063.

Among patients with insufficient consolidation (LSS < 3) after 2 years, 5 of 11 patients (45.5%) improved to sufficient consolidation (LSS ≥ 3) at the 10-year follow-up. In particular, 5 out of 7 patients (71.4%) with an LSS of 2 after 2 years showed further improvement to LSS ≥ 3 after 10 years.

Among the six patients with further insufficient consolidation after 10 years, one patient ultimately underwent amputation several years after the index procedure and was therefore assigned an LSS of 0 at the 10-year follow-up. Two additional patients required revision surgery because of treatment failure. Although both achieved osseus consolidation following revision, they were likewise assigned an LSS of 0 according to the study protocol.

In one patient, bony union could not be achieved because of severe comorbidities and chronic osteomyelitis; however, the retained intramedullary nail provided sufficient stability for limited ambulation. Two further patients developed stable pseudarthroses without pain or radiographic progression. As both remained clinically asymptomatic, no further surgical intervention was indicated.

The median LSS after ten years was 4 (IQR: 3–4). An LSS of 4 was observed in 38 patients (70.4%), and 10 additional patients (18.5%) achieved an LSS of 3. Thus, 48 patients (88.9%) demonstrated radiological evidence of stable osseous consolidation at a time point after ten years. The remaining 6 patients (11.1%) had an LSS of ≤ 2. Among these, 4 patients required revision surgery after the index procedure and were classified as treatment failures; consequently, they were assigned an LSS value of 0.

**Fig. 3:**
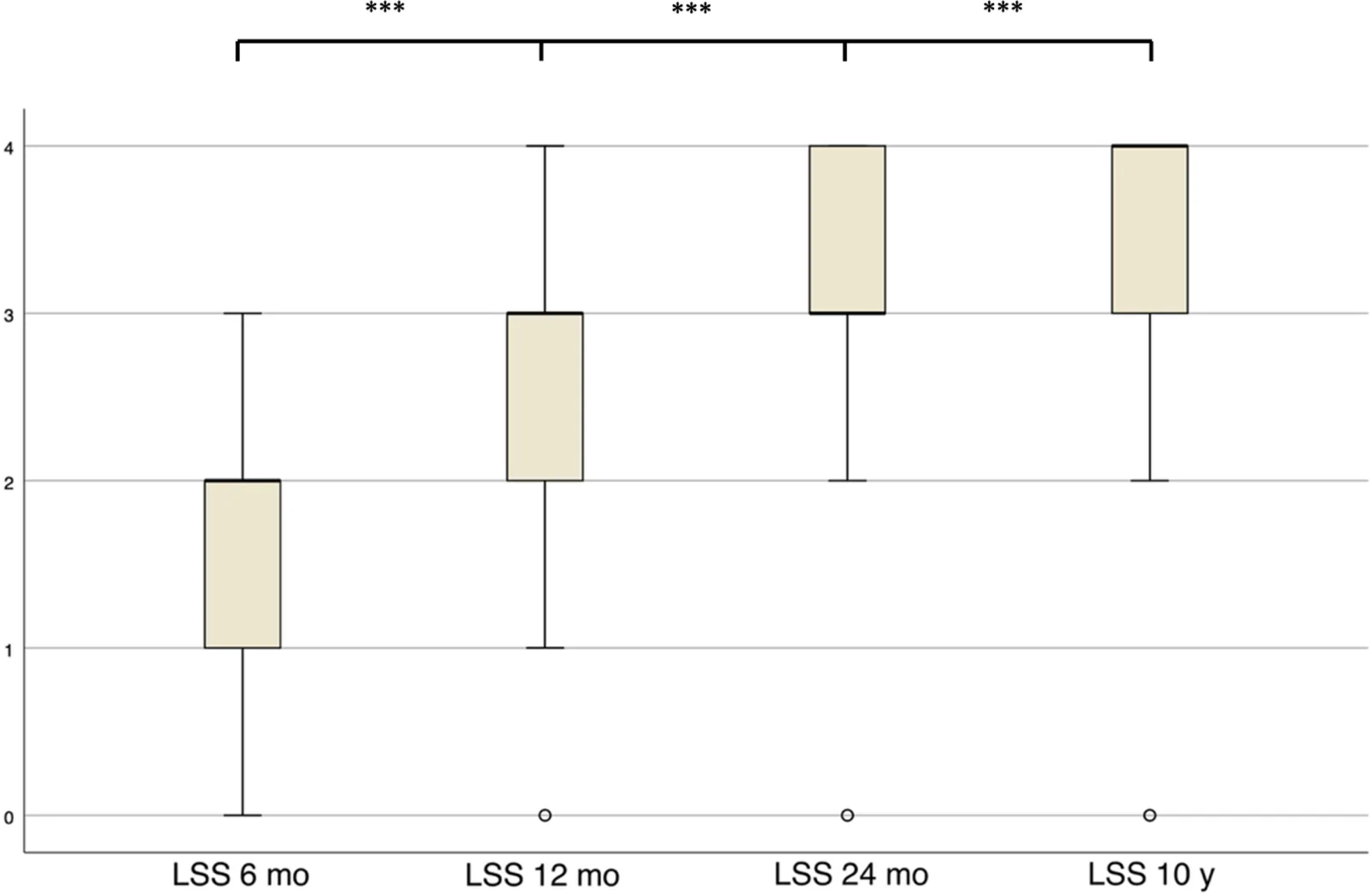
Box plot of Lane-Sandhu-Score (LSS) values at the respective points in time with median, IQR and dispersion

Figure 3 illustrates the distribution of LSS values at the four follow up time points.

**Fig. 4:**
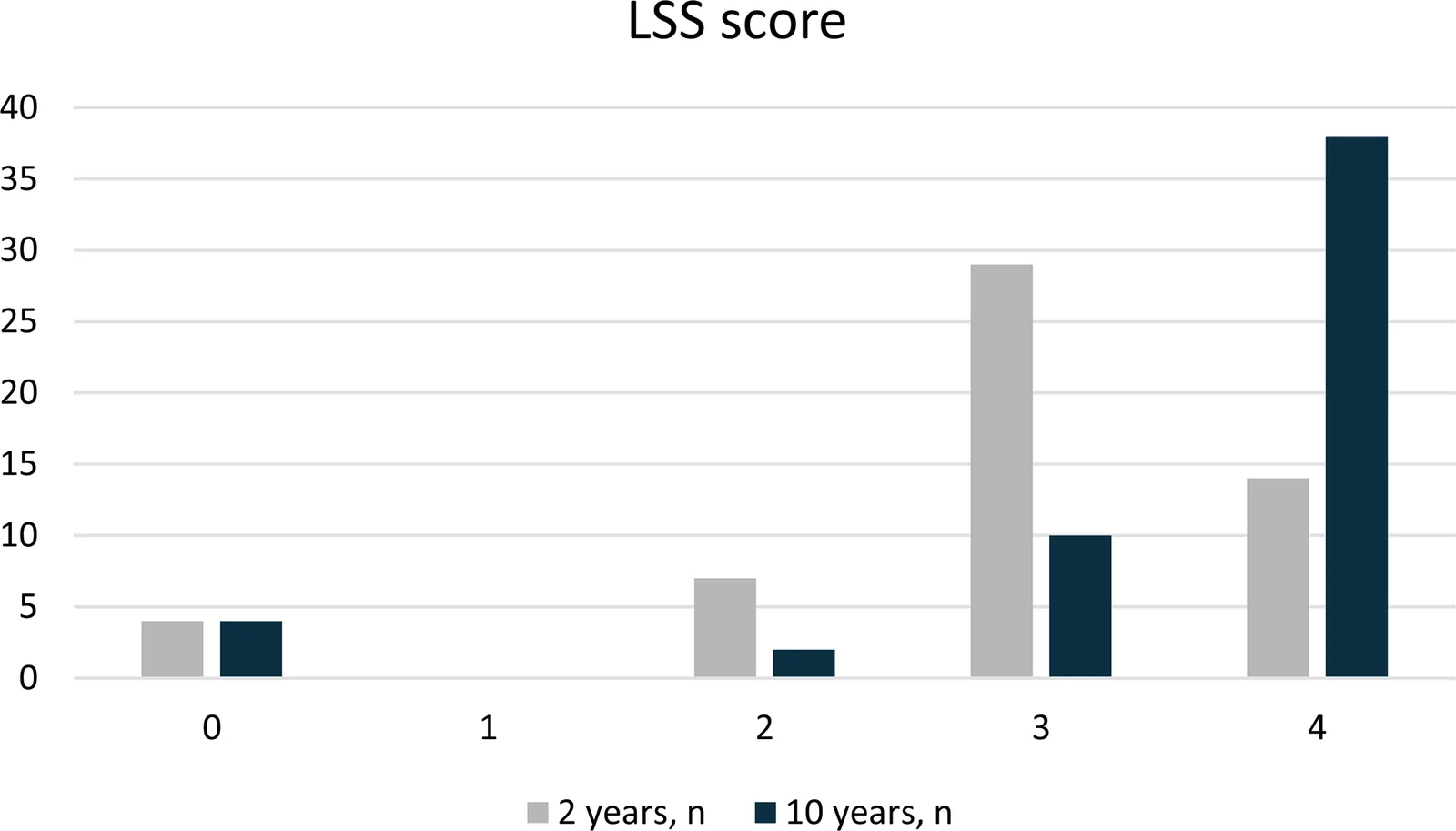
Comparison of LSS-score achieved after two years and ten years

A progressive increase in LSS over time was observed. The median LSS was 2 (IQR: 1–2) at 6 months, 3 (IQR: 2–3) at 12 months, 3 (IQR: 3–4) at 24 months, and 4 (IQR: 3–4) at 10 years. Pairwise comparisons between consecutive follow-up time points using the Wilcoxon signed-rank test demonstrated significant differences in LSS at each reminding interval (*p* < 0.001 for all comparisons). Figure 4 illustrates the achieved LSS-Score after two and ten years.

A statistically significant difference was observed after two and 10 years (Z = 4.716, *p* < 0.001) using the Wilcoxon sing-rank test. The distribution of LSS values at 2 and 10 years is shown in Table 2.

**Table 2:** Lane-Sandhu-Score (LSS) distribution after 2 years and 10 years

LSS	2 years, *n* (%)	10 years, *n* (%)
0	4 (7,4)	4 (7,4)
1	0 (0,0)	0 (0,0)
2	7 (13,0)	2 (3,7)
3	29 (53,7)	10 (18,5)
4	14 (25,9)	38 (70,4)

**Fig. 5:**
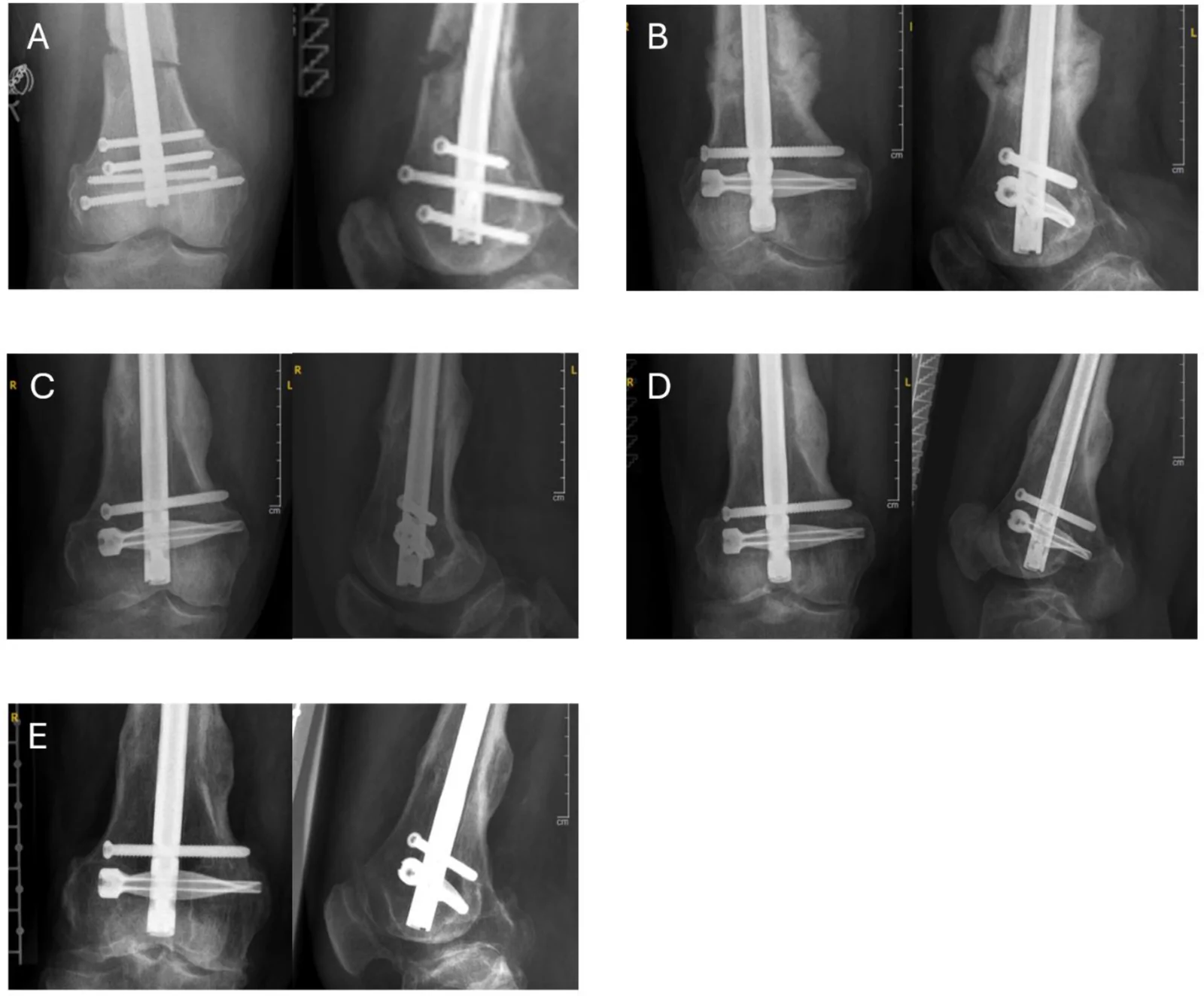
Example 1 of a case with a femur non-union during a 10-year follow-up. (**A**) Preoperative X-ray (**B**) Postoperative X-ray after 6 months. (**C**) Postoperative X-ray after 12 months. (**D**) Postoperative X-ray after 24 months with full osseus consolidation. (**E**) Postoperative X-ray after ten years with still ongoing remodeling

**Fig. 6:**
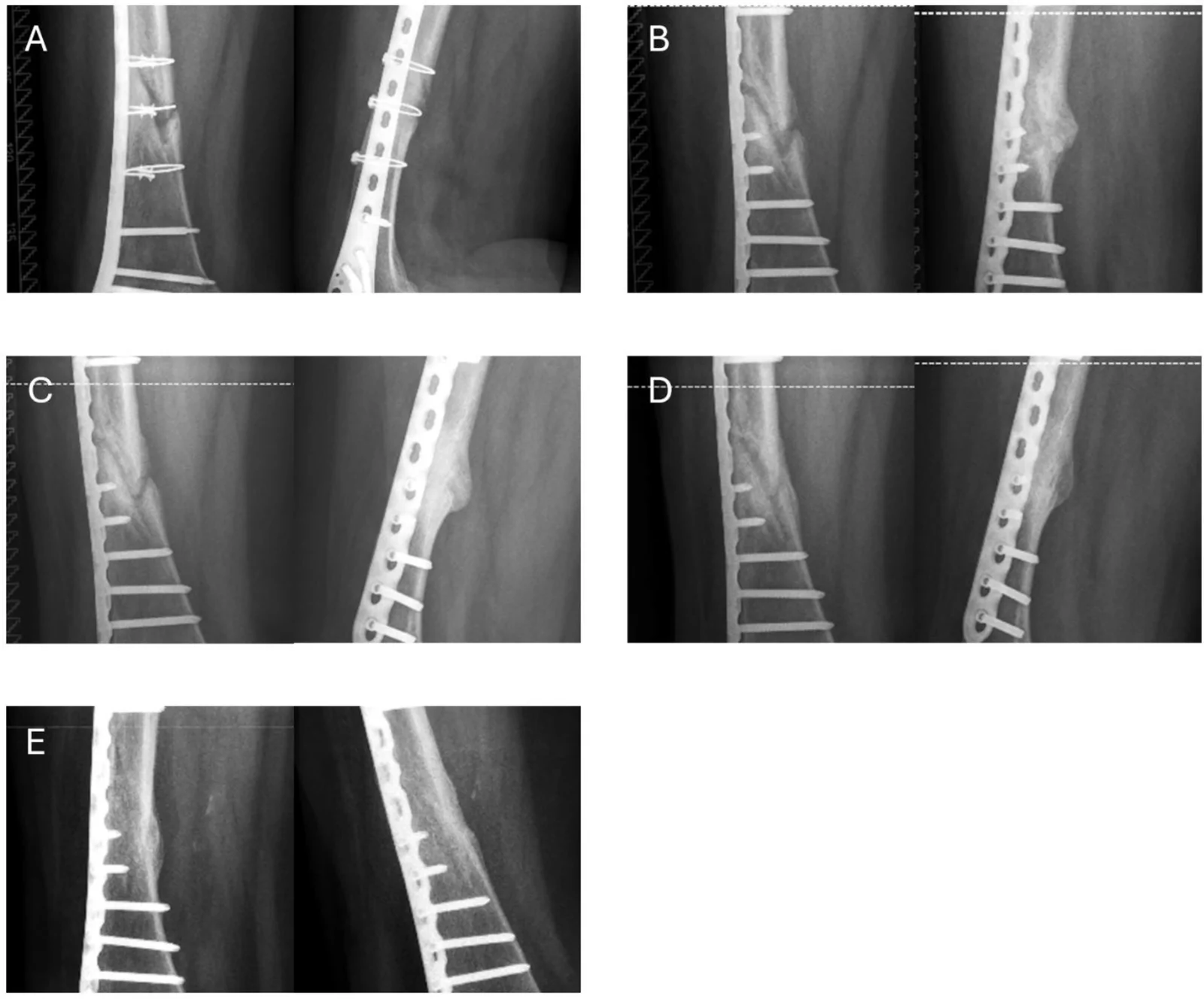
Example 2 of a case with a femur non-union during a 10-year follow-up. (**A**) Preoperative X-ray (**B**) Postoperative X-ray after 6 months. (**C**) Postoperative X-ray after 12 months. (**D**) Postoperative X-ray after 24 months with full osseus consolidation with a LSS of 3. (**E**) Postoperative X-ray after ten years with fill osseus consolidation (LSS 4)

Figures 5 and 6 show a distal femoral non-union from our study population. It can be seen that bone remodeling takes place throughout the entire follow-up period.

### Clinical outcomes

At ten-year follow-up, 51 patients (94.4%) reported an overall satisfaction with the treatment.

Among 46 patients with femoral or tibial involvement, 12-month postoperative knee range of motion (ROM) data were available for 15 patients. Median knee ROM was 130° (IQR 120°–135°) at 12 months and 130° (IQR 120°–135°) at 10 years. Using the minimum clinically important difference (MCID) for knee ROM defined by Guzik et al. (8.48°) [13], six patients (40%) showed a clinically relevant change. ROM increased by a mean of 41.7° in 3 patients (20%) and decreased by a mean of 26.7° in 3 patients (20%). The remaining 9 patients (60%) showed no change exceeding the MCID threshold.

A subjective strength deficit of the affected limb compared with the contralateral side 10 years after the non-union therapy was reported by 70.4% of patients, whereas 29.6% reported no strength difference.

Among 47 patients with lower extremity involvement including also one patient with non-union of the metatarsal bone, 33 (70.2%) reported a maximum walking distance ≥ 500 m after ten years. Four patients (8.5%) were able to walk up to 500 m, three (6.4%) up to 200 m, four (8.5%) up to 100 m, and two (4.3%) up to 50 m; 1 patient reported inability to walk.

Return to previous employment was reported by 68.5% of patients; the remaining individuals had either retired early (*n* = 12) or reached regular retirement age (*n* = 6).

After ten years, the mean SF-12 scores for the 54 study participants were 53.14 ± 10.13 for the mental health component (MCS) and 38.93 ± 12.71 for the physical health component (PCS). Twelve-month postoperative data were available for 20 patients, with mean MCS of 48.23 ± 10.37 and PCS of 40.16 ± 10.26.

No statistically significant differences were observed between 12 months and 10 years (MCS: mean difference − 4.72 ± 12.77, *p* = 0.115; PCS: mean difference 1.23 ± 15.03, *p* = 0.718).

At ten-year follow-up, patients had a median VAS score of 3.25 (IQR: 2.25–6.25). Out of the 54 patients, 13 patients reported a pain score of 0. At twelve months postoperatively, the median VAS score was 5.00 (IQR: 1.25–6.00).

Between these two timepoints no significant difference could be found with *n* = 20 (Z = − 0.15, *p* = 0.881).

A similar picture emerged regarding the frequency of pain: 38.9% of respondents rarely experienced pain, 24.1% occasionally, another 24.1% regularly, and 13.0% reported feeling pain constantly.

More than half of the patients (51.9%) stated that they did not need any pain medication at the timepoint of the follow-up. About one-third (29.6%) stated that they took painkillers occasionally, while 18.5% were regularly dependent on analgesics.

#### Influence of risk factors

Univariable analyses revealed no significant associations between smoking exposure, obesity, or diabetes mellitus and postoperative functional or consolidation and patient-reported outcomes, including LSS, SF-12 physical and mental component scores, and VAS (Tables 3, 4 and 5).

**Table 3:** Association between smoking exposure and postoperative outcomes

Outcome	≤ 6.1 pack-years (*n* = 28)	> 6.1 pack-years (*n* = 26)	*p*-value
LSS	4 (4–4)	4 (3–4)	0.161
SF-12 PCS	39.1 (28.15–52.10)	41.25 (28.88–53.05)	0.775
SF-12 MCS	54.2 (42.0–58.48)	53.7 (37.08–58.98)	0.829
VAS	4.25 (0.25–6.88)	3.50 (1.50–6.13)	0.496

Values are presented as median (interquartile range). LSS, Lower Extremity Functional Scale; SF-12 PCS, physical component summary score; SF-12 MCS, mental component summary score; VAS, visual analogue scale

**Table 4:** Association between obesity and postoperative outcomes

Outcome	Non-obese patients (*n* = 32)	Obese patients (*n* = 22)	*p*-value
LSS	4 (3–4)	4 (3.75–4)	0.273
SF-12 PCS	42.5 (29.18–54.1)	38.75 (24.1–50.83)	0.218
SF-12 MCS	55.4 (42.23–58.6)	53.95 (39.28–58.95)	0.840
VAS	3.75 (0.25–6.0)	3.75 (1.5–6.5)	0.600

Values are presented as median (interquartile range). LSS, Lower Extremity Functional Scale; SF-12 PCS, physical component summary score; SF-12 MCS, mental component summary score; VAS, visual analogue scale

**Table 5:** Association between diabetes and postoperative outcomes

Outcome	Non-diabetic patients (*N* = 42)	Diabetic patients (*n* = 12)	*p*-value
LSS	4 (3–4)	4 (4–4)	0.214
SF-12 PCS	43.1 (28.9–53.2)	35.55 (23.08–41.95)	0.109
SF-12 MCS	53.95 (39.8–58.2)	55.05 (41.48–60.73)	0.693
VAS	3.5 (0.75–6.13)	5.0 (0.5–6.88)	0.431

Values are presented as median (interquartile range). LSS, Lower Extremity Functional Scale; SF-12 PCS, physical component summary score; SF-12 MCS, mental component summary score; VAS, visual analogue scale

The multivariable logistic regression model was statistically significant overall (LR χ²(3) = 8.67, p = 0.0341; pseudo R² = 0.2300). Infection was independently associated with lower odds of consolidation (OR 0.04, 95% CI 0.003–0.58; p = 0.018), whereas BMI and smoking exposure were not significantly associated with consolidation (Table 6). Diabetes mellitus was excluded from the model because of collinearity

**Table 6:** Multivariable logistic regression analysis of factors associated with consolidation

Variable	Odds ratio (OR)	95% Confidence interval	*p*-value
BMI	3.56	0.18–69.23	0.402
Infection	0.04	0.003–0.58	0.018
Smoking exposure (> 6.1 pack-years)	1.69	0.24–12.02	0.600

Model characteristics: Number of observations = 54; LR χ² = 8.67; *p* = 0.0341; pseudo R² = 0.2300

#### Post-operative complications

Of the 54 patients included, four had to undergo revision surgery due to screw breakage (*n* = 1) and recurrent infections (*n* = 3). After several years without satisfactory consolidation and following detailed consultation, one patient opted for a lower leg amputation.

## Discussion

The aim of the present study was to evaluate long-term outcomes of patients treated surgically for non-union, with a particular focus on osseous consolidation, pain, range of motion, and health-related quality of life ten years after surgery. In addition, outcomes were compared with data obtained from the same cohort at earlier follow-up time points.

A continuous increase in LSS values over time suggests ongoing long-term remodeling even years after surgical treatment of non-union. This observation is consistent with the known biological potential of bone for prolonged remodeling following fracture healing and non-union treatment. Despite this radiological improvement, no significant changes were observed in clinical outcomes such as pain, range of motion, or health-related quality of life between earlier follow-up time points and ten years. One possible explanation is that the transition from LSS 3 to LSS 4 represents primarily a radiological refinement rather than a clinically perceptible improvement. Accordingly, although a progressive increase in LSS was observed, the proportion of consolidated patients did not change significantly between two and ten years, which is consistent with the stable clinical outcomes. These findings suggest that healed non-unions continue to undergo long-term remodeling, reflecting sustained osteogenic activity and biological vitality of bone, even in the absence of measurable clinical improvement.

From a clinical perspective, these findings may have important implications for decision-making at the two-year follow-up. Despite the fact that incomplete radiographic consolidation at this stage may lead to concerns regarding treatment success, our long-term results indicate that delayed bone healing can continue beyond two years and may ultimately provide sufficient stability. Consequently, the absence of complete consolidation at two years should not be taken as an indication for revision surgery in clinically stable patients. It is posited that a favorable long-term outcome may be expected in cases where fracture alignment and implant stability are maintained, progressive radiographic signs of bone formation are present, and patients are able to achieve full weight-bearing without pain or functional deterioration. Conversely, persistent pain, mechanical symptoms, lack of radiographic progression, loss of alignment, or signs of implant-related complications may indicate a higher risk of treatment failure and the potential need for revision surgery. While several studies have investigated consolidation rates and clinical outcomes following non-union surgery, long-term data extending to a decade after non-union treatment remain scarce. The consolidation rate observed in the present study is comparable to previously published results, despite the inclusion of complex cases with infected non-unions and long-term follow-up. Previous studies have reported high consolidation rates following surgical treatment of long-bone non-unions, ranging from approximately 77% to 98%, depending on patient characteristics and treatment strategies [14–16]. Higher consolidation rates have been described in cohorts treated according to the Diamond Concept; however, infected non-unions were excluded in this study, whereas approximately one-third of patients in our cohort presented with infection, representing a more complex patient population [14]. Other studies including mixed cohorts of femoral and tibial non-unions reported consolidation rates of approximately 90%, which is consistent with the 88.9% consolidation rate observed in our cohort at ten years [15, 16]. Overall, the consolidation rate observed in the present study is within the range of previously reported outcomes, despite the inclusion of infected non-unions and the extended follow-up period.

With regard to health-related quality of life, SF-12 scores at ten years remained largely unchanged compared to values obtained one year postoperatively. Mean SF-12 mental component scores were comparable to German population norms, whereas physical component scores remained substantially lower, indicating preserved mental health-related quality of life but persistent limitations in physical function [17]. Previous studies evaluating long-term outcomes after non-union treatment have similarly reported persistent limitations in physical function and health-related quality of life after surgical treatment [8, 18, 19].

When interpreting these findings, the expected age-related decline in physical SF-12 scores in the general population should be considered. Thus, the absence of a decline in physical SF-12 scores over a ten-year period may indicate a favorable long-term outcome, as a reduction in physical health-related quality of life would generally be expected with increasing age [17, 20–22].

Range of motion did not show significant improvement between one and ten years postoperatively, suggesting stabilization of joint mobility after the first postoperative year. This finding is consistent with the results of Boadi et al., who reported no clear long-term improvement or deterioration in joint mobility after surgical treatment of aseptic non-unions at a mean follow-up of eight years [23]. Together, these data suggest that functional recovery in terms of joint mobility occurs primarily within the first postoperative year.

Interestingly, established risk factors such as smoking did not significantly influence long-term clinical outcomes in our cohort. This finding contrasts with previous studies identifying smoking as a negative predictor of bone healing [24, 25]. However, given the limited sample size, particularly in subgroup analyses, this result should be interpreted with caution.

Several limitations of this study must be acknowledged. First, complete longitudinal data on range of motion and SF-12 scores were available only for a limited number of patients. Additionally, changes in comorbidities, socioeconomic status or age-related health decline over the ten-year period may have influenced patient-reported outcomes. Radiological consolidation was assessed exclusively using plain radiographs in two planes, which is less sensitive than advanced imaging modalities such as computed tomography. Finally, outcomes were analyzed across all non-union locations, potentially obscuring location-specific differences.

Despite these limitations, the present study provides valuable insights into the long-term radiological and clinical outcomes after surgical treatment of non-union and demonstrates sustained consolidation and stable patient-reported outcomes up to ten years postoperatively.

## Conclusion

Surgical treatment of non-union resulted in sustained osseous consolidation and stable clinical outcomes at ten-year follow-up in the majority of patients. Radiological remodeling continued to improve over time, while patient-reported outcomes, including pain, mobility, and quality of life, remained largely unchanged after the first postoperative year. Despite persistent limitations in physical function, long-term patient satisfaction was high. These findings suggest that successful non-union treatment provides durable long-term results, although functional recovery might plateau early.

## Data Availability

The datasets used and/or analysed during the current study are available from the corresponding author on reasonable request.
